# Plasma Exosomes Spread and Cluster Around β-Amyloid Plaques in an Animal Model of Alzheimer’s Disease

**DOI:** 10.3389/fnagi.2017.00012

**Published:** 2017-02-01

**Authors:** Tingting Zheng, Jiali Pu, Yanxing Chen, Yanfang Mao, Zhangyu Guo, Hongyu Pan, Ling Zhang, Heng Zhang, Binggui Sun, Baorong Zhang

**Affiliations:** ^1^Department of Neurology, Second Affiliated Hospital, School of Medicine, Zhejiang UniversityHangzhou, China; ^2^Department of Neurobiology, School of Basic Medical Sciences, Key Laboratory of Medical Neurobiology (Ministry of Health of China), Key Laboratory of Neurobiology of Zhejiang Province, Zhejiang University School of MedicineHangzhou, China

**Keywords:** exosomes, Alzheimer’s disease, microglia, aging, β-amyloid plaques

## Abstract

Exosomes, a type of extracellular vesicle, have been shown to be involved in many disorders, including Alzheimer’s disease (AD). Exosomes may contribute to the spread of misfolded proteins such as amyloid-β (Aβ) and α-synuclein. However, the specific diffusion process of exosomes and their final destination in brain are still unclear. In the present study, we isolated exosomes from peripheral plasma and injected them into the hippocampus of an AD mouse model, and investigated exosome diffusion. We found that injected exosomes can spread from the dentate gyrus (DG) to other regions of hippocampus and to the cortex. Exosomes targeted microglia preferentially; this phenomenon is stable and is not affected by age. In AD mice, microglia take up lower levels of exosomes. More interestingly, plasma exosomes cluster around the Aβ plaques and are engulfed by activated microglia nearby. Our data indicate that exosomes can diffuse throughout the brain and may play a role in the dynamics of amyloid deposition in AD through microglia.

## Introduction

Exosomes are a bioactive vesicle (40–100 nm) secreted by various cells *in vitro* and *in vivo* under physiological and pathological conditions. They have been isolated from biological fluids such as blood, cerebrospinal fluid and urine (Pisitkun et al., 2004; Caby et al., 2005; Vella et al., 2008). A number of proteins related to neurodegenerative disorders, including prion disease, Parkinson’s disease and Alzheimer’s disease (AD), have been shown to be released by cells in association with exosomes (Coleman and Hill, 2015). Evidence demonstrates that exosomes can facilitate the unique transmissible nature of prions (Fevrier et al., 2004; Vella et al., 2007; Guo et al., 2016); the exosomes from human-derived prions infected cells are efficient initiators of prion propagation in uninfected recipient cells and produce clinical prion disease when inoculated into mice (Vella et al., 2007; Guo et al., 2016). While prion disease has traditionally been thought to be the only neurodegenerative disease that is transmissible, misfolded forms of key proteins involved in other neurodegenerative disorders such as AD, Parkinson’s disease and amyotrophic lateral sclerosis, may also spread in a similar way (Bellingham et al., 2012; Coleman and Hill, 2015).

AD, the most common type of dementia, is pathologically characterized by extracellular deposition of senile plaques and intracellular accumulation of neurofibrillary tangles (Kandimalla et al., 2016a,b; Manczak et al., 2016). A growing number of studies suggest that AD, including amyloid deposits and neurofibrillary tangles, develops in a prion-like manner (Eisele et al., 2009; Frost and Diamond, 2010; Coleman and Hill, 2015). Experimental seeding of amyloid-β (Aβ) pathology has been shown in primates and transgenic mice by intracerebral or peripheral inoculation with AD brain homogenate (Baker et al., 1994; Meyer-Luehmann et al., 2006; Eisele et al., 2010; Hamaguchi et al., 2012; Heilbronner et al., 2013). An autopsy study also found marked deposition of gray matter and vascular Aβ in relatively young patients with iatrogenic Creutzfeldt–Jakob disease (Jaunmuktane et al., 2015). Therefore, exosomes have been suspected to participate in the prion-like propagation of lesions in AD (Bellingham et al., 2012; Vingtdeux et al., 2012; Coleman and Hill, 2015). This idea is supported by the observation that exosomes isolated from either neuronal cell cultures or brain contain Aβ precursor protein (APP) and APP-processing products, including C-terminal fragments and Aβ (Vingtdeux et al., 2007; Perez-Gonzalez et al., 2012). Moreover, exosomes can cross the blood–brain barrier and are exploited as drug delivery vehicles in many studies (Alvarez-Erviti et al., 2011; Haney et al., 2015). Exosomes contribute to the long distance transmission of biological information and therefore affect many physiological and pathological processes. However, the specific diffusion process of exosomes in the brain is still unclear. In addition, it is unknown if the peripheral circulation can communicate with the central nervous system via exosomes.

Microglia, the resident immune cells in the brain, phagocytose dead cells and help to clear misfolded protein aggregates such as amyloid plaques in AD (Bard et al., 2003). *In vitro* studies show that neuron-derived and oligodendrocyte-derived exosomes are incorporated into microglia (Fitzner et al., 2011; Yuyama et al., 2012). It is unknown, however, whether microglia are also the final destination of peripheral exosomes. Exosomes from brain can enter the bloodstream and have been explored as potential biomarkers of preclinical AD or other neurodegenerative diseases (Shi et al., 2014; Fiandaca et al., 2015; Goetzl et al., 2015). Exosomes are reported to dramatically stimulate Aβ fibril formation by its surface glycosphingolipids, and mediate Aβ fibrils uptake into microglia in a phosphatidylserine-dependent manner (Yuyama et al., 2012). Intracerebrally injected exosomes result in reduction of Aβ pathology (Yuyama et al., 2014, 2015). Secretion of exosomes is decreased in progranulin-associated frontotemporal dementia (Benussi et al., 2016). However, Dinkins et al. (2014) show that exosomes interfere with the uptake of Aβ by primary cultured astrocytes and microglia *in vitro*. Preventing exosome secretion with GW4869 can reduce amyloid plaque formation *in vivo*. Conversely, increasing the secretion of exosomes enhances plaque formation (Dinkins et al., 2014, 2015). Furthermore, reducing exosome secretion by genetic neutral sphingomyelinase-2 defects in 5XFAD mice ameliorates AD-associated pathology and improves cognition (Dinkins et al., 2016). Further studies are needed to explore the role of exosomes in AD pathology, especially in amyloid deposition.

In the present study, we traced the spread of exogenous plasma exosomes to explore the specific diffusion processes of exosomes in mouse brain and their target cells. In addition, we investigated the role of exosomes in amyloid deposition in an AD transgenic mouse model.

## Materials and Methods

### Animals

hAPP-J20 mice expressing human APP with Swedish and Indiana (KM670/671NL, V717F) mutations were purchased from the JAX MMRRC (Stock # 034836). They were housed in groups of five under a normal 12 h light/dark cycle, kept under standard temperature and humidity and in pathogen free conditions. All experiments were approved by the Institutional Animal Care and Use Committee of Zhejiang University and carried out in accordance with the National Institutes of Health guidelines for the use of laboratory animals.

### Cell Cultures

The human neuroblastoma cell line SH-SY5Y (SY5Y) expressing GFP was cultured using Dulbecco’s modified Eagles medium (DMEM) supplemented with 10% fetal bovine serum in a 5% CO2 humidified incubator at 37°C.

### Blood Collection and Platelet Free Plasma (PFP) Preparation

We collected blood from hearts of 2 months old C57BL/6 mice, then prepared platelet free plasma (PFP) as described previously (György et al., 2014; Osteikoetxea et al., 2015) and in accordance with the standardization of International Society on Thrombosis and Hemostasis on blood sampling and handling for MV analysis (Lacroix et al., 2013). Briefly, in order to prevent release of platelet-derived EVs *in vitro*, blood was collected into acid-citrate-dextrose tubes (Greiner Bio-One; György et al., 2014). Blood was centrifuged (2 × 15 min, 3000 g at 4°C). PFP was stored at −80°C until use.

### Exosome Isolation

We isolated exosomes from PFP using total exosome isolation reagents (Life Technologies). Vesicles were enriched according to the manufacturers’ instructions. Briefly, the plasma sample was centrifuged (20 min, 2000× g; 20 min, 10,000× g at room temperature) to remove cells and debris. The supernatant was transferred to a new tube, then 0.5 volumes of 1× PBS were added and mixed, before adding 0.05 volumes of Proteinase K, followed by 10 min incubation at 37°C. Then 0.2 volumes of exosome precipitation reagent was added to the sample following by incubation at 4°C for 30 min. After incubation, the sample was centrifuged at 10,000× g for 30 min at room temperature. Exosomes were contained in a pellet at the bottom of the tube.

### Electron Microscopy

Exosomes were resuspended in PBS at a concentration of 100 μg protein/ml. The exosome mixture was then applied to the grid and negatively stained with 2% phosphotungstic acid. Transmission images were acquired using an HT-7700 transmission electron microscope (Hitachi, Tokyo, Japan).

### Nanoparticle Tracking Analysis

To quantify exosomes and characterize their dispersion and size distribution, nanoparticle tracking analysis (NTA) was performed using a NanoSight LM10 instrument (Malvern, Instruments) and NTA Version 2.3 Build 0034 software. For NTA, 100 μl of exosomes were diluted in 50 ml PBS, and particle size was calculated automatically for at least 5000 particles. PBS was assessed before the experiment to ensure that it was particle-free.

### Western Blotting Analysis

Exosome samples were subjected to SDS-PAGE on 12% gels, followed by Western blotting. Proteins were blotted to PVDF membranes, and membranes were blocked in 5% milk in PBS/0.1% Tween 20 (PBS/Tween). The following primary antibodies were used: rabbit anti-ALIX (1:1000, Abcam, ab88388), rabbit anti-Tsg101 (1:2000, Abcam, ab125011), rabbit anti-GM130 (1:1000, ABclonal, A5344). Blots were visualized using HRP-conjugated secondary antibodies and the ECL Detection Reagent (Thermo Fisher Scientific) and were imaged on a LAS3000 image reader (Raytest, Germany).

### Exosome Tracking

Exosomes were labeled with a red fluorescent lipophilic dye DiI (1,1′-dioctadecyl-3,3,3′,3′-tetramethylindocarbocyanine perchlorate; Beyotime, C1036), allowing monitoring of exosome movement. DiI emits strong fluorescence when excited by green light and incorporated into membranes, and does not disrupt the membrane properties. Exosomes were resuspended in sterile PBS and incubated with 5 μM DiI for 10 min. DiI-exosomes were then washed and resuspended in sterile PBS three times to remove free DiI and other impurities such as lipoproteins. The control was prepared by DiI incubation with PBS, which was washed as for the DiI-exosome preparation.

### Cell Incubation with DiI-Exosomes

DiI-exosomes were administered to SH-SY5Y cells and incubated for 24 h in serum-free conditions at a working concentration of 2 μg protein/ml. Images were acquired using an inverted microscope (Olympus, IX53).

### Stereotaxic Injection of Exosomes into the Mouse Hippocampus

The DiI-exosome solution (3 μl at 1 μg protein/μl) and the control (3 μl) was stereotactically injected into the dentate gyrus (DG) of the mouse hippocampus using a stereotactic apparatus (KOPF), at the following coordinates (relative to Bregma): anterior posterior: −2.0, medial lateral: ± 1.6, dorsal ventral: −2.1. The brains were perfused with cold 0.9% saline and fixed in 4% paraformaldehyde for 3–20 days after injection.

### Immunofluorescence Staining

Mice were perfused transcardially with 0.9% saline. Brains were removed immediately and immersed in 4% paraformaldehyde. After dehydration in 30% sucrose, coronal brain sections (20 μm) were prepared with a sliding microtome (Leica). Brain slices were stained as previously described (Zheng et al., 2015). In brief, brain slices were blocked with blocking buffer (10% fetal bovine serum, 1% nonfat milk, 0.2% gelatin in PBS containing 0.5% Triton X-100) and incubated with primary antibodies: rabbit anti-MAP-2 (1:600, #4542, CST), rabbit anti-IBA1 (1:600, 019-19741 Wako), mouse anti-6E10 (1:1000, SIG-39320, Covance), monoclonal anti-glial fibrillary acidic protein (GFAP, 1:400, G3893, Sigma). After incubating with primary antibodies overnight at 4°C, the brain sections were washed three times with PBS containing 0.5% Triton X-100 and three times with PBS. Slices were then incubated with the appropriate secondary antibodies: AlexaFluor 488 AffiniPure donkey anti-rabbit lgG (1:300, 711-545-152, Jackson ImmunoResearch), AlexaFluor 647 donkey anti-rabbit (1:1000, A-31571, Invitrogen), AlexaFluor 488 donkey anti-mouse (1:1000, A-21206, Invitrogen). After incubation with secondary antibodies for 2 h at room temperature, the brain sections were washed once with PBS containing 0.5% Triton X-100 and three times with PBS. Free floating brain sections were mounted on glass slides with a drop of 4′,6-diamidino-2-phenylindole (DAPI) Fluoromount-G mounting medium (0100-20, Southern Biotech). Images were taken using confocal microscope (OLYMPUS FV1000) and a large field of view CCD system (Zeiss, Axio Scan.Z1). Then images were processed using Fluoview Viewer (FV10-ASW; Olympus) and the ZEN 2 (blue edition, V1.0 en; Zeiss) software packages, respectively.

### Statistical Analysis

Statistical analyses were performed using GraphPad Prism version 6.05 for Windows (GraphPad Software, San Diego, CA, USA). All values in the figures are presented as mean ± standard error of the mean (SEM). Differences between two means were assessed with unpaired two-tailed Student’s *t* test. *P* values of less than 0.05 were considered statistically significant.

## Results

### Characterization of Exosomes

**Figure 1:**
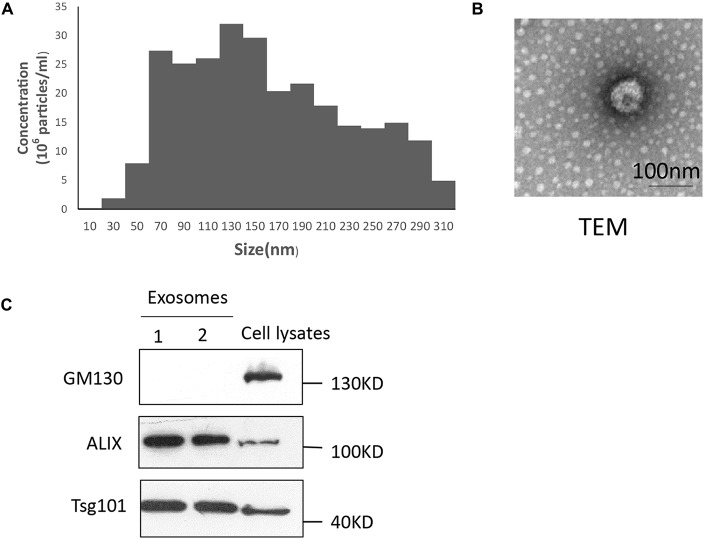
**Characterization of plasma exosomes. (A)** Exosomes were measured via nanoparticle tracking analysis (NTA) analysis to quantify exosomes and show their size distribution. Exosomes comprise a homogenous population with a peak in diameter at 137 nm and low polydispersity. **(B)** Exosomes (1,00,000× g pellet) underwent negative staining with phosphotungstic acid and were examined by electron microscopy. Scale bars, 100 nm. **(C)** Fractions of exosomes were analyzed by Western blotting to detect the exosomal markers Alix and Tsg101, and the negative marker GM130. All approximate protein masses are represented in kDa.

Exosomes were analyzed for morphology and size distribution using a Nanosight system and transmission electron microscope, respectively (Figures 1A,B). Consistent with recent reports (Momen-Heravi et al., 2012; Chernyshev et al., 2015), the exosomes were observed as a homogenous population with low dispersity and with a peak in particle size at 137 nm (Figure 1A). Streaming, a factor related to Brownian motion of small particles, caused the reported size distribution to be larger than actual size distribution (Scott et al., 2005). Transmission electron microscopy revealed that the media contain morphologically distinct particles of approximately 60–110 nm diameter that are membrane bound and “cup shaped” (Figure 1B) as previously described. The presence of exosomes was confirmed by detecting the exosomal markers Alix and Tsg101, and negative marker GM130 in Western blotting analysis (Figure 1C).

### Exosomes Can Diffuse Over Great Distances in Mouse Brain

**Figure 2:**
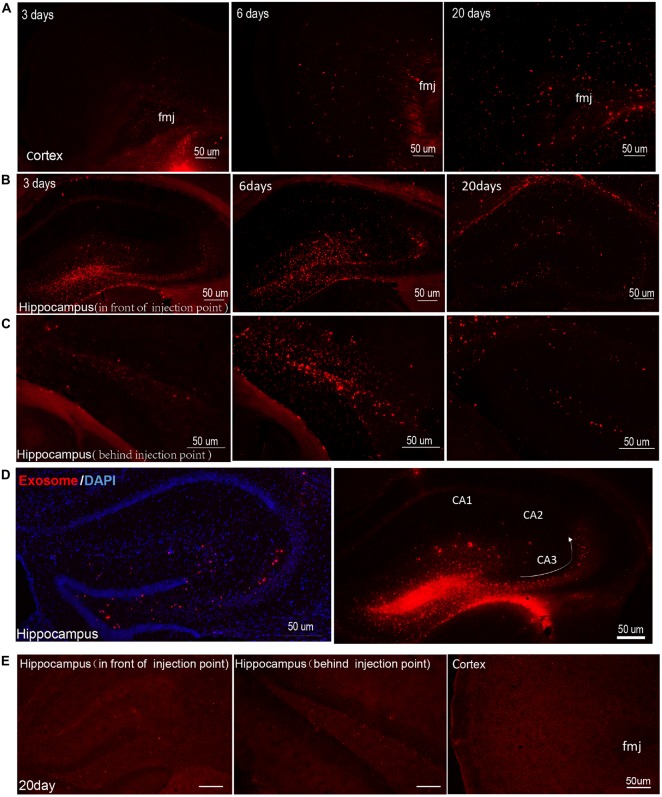
**Exosomes diffuse over great distances in mouse brain.** Images were captured at 3, 6 and 20 days after injection of DiI-stained exosomes into the hippocampal dentate gyrus (DG) region. DiI-exosomes can emit red fluorescence. **(A)** Representative photomicrographs showing the distribution of exosomes in cortex. In order to show the structure more clearly, we marked the forceps major corpus callosum (fmj) in figures. The content of exosomes increased with time in cortex. **(B,C)** Representative photomicrographs showing the distribution of exosomes in hippocampus in front of **(B)** and behind **(C)** the injection point. Exosomes show better diffusion in hippocampus on day 6 than on day 3. On the 20th day, most of the exosomes spread from the hippocampus to the cortex. **(D)** Images were captured 3 days after injection of exosomes in hippocampal DG region. The figure on the left show DiI-exosomes and counterstain with 4′,6-diamidino-2-phenylindole (DAPI), while the figure on the right shows DiI-exosomes only. The arrow shows the projection direction from the DG region to CA3. Exosomes concentrated in hippocampal CA3 region. In contrast, very few exosomes are observed in area CA1 and CA2 of the hippocampus. **(E)** The control (DiI incubation with PBS and being re-isolated, washed, resuspended in sterile PBS for three times as the DiI-exosomes’ preparation) were injected into hippocampal DG region. Representative photomicrographs show images captured at 20 days after injection and the images captured at 3 and 6 days after injection are shown in Supplementary Figure S2. Scale bars, 50 μm.

Several reports have revealed that exosomes facilitate the unique transmissible nature of prions (Vella et al., 2007; Guo et al., 2016). Key proteins involved in other neurodegenerative disorders such as AD, Parkinson’s disease and amyotrophic lateral sclerosis may also spread via misfolded proteins similar to prions (Bellingham et al., 2012; Coleman and Hill, 2015). To identify the trail of exosomes in the brain, we injected DiI-labeled exosomes into the mouse DG and traced the movement of the exosomes over time (3, 6 and 20 days after injection). We found that exosomes could spread from the injection site to other areas of hippocampus gradually and moved to the cortex as time went on (Figures 2A–C). Fluorescent images showed the most abundant exosomes in the cortex on day 20, but none were observed on day 3; a small portion of exosomes began to appear in the cortex on the 6th day after injection (Figure 2A). On the 20th day, most of the exosomes had spread from hippocampus to the cortex and only a few exosomes were retained in the hippocampus (Figures 2B,C). We prepared 15 coronal brain sections at approximately 200 μm intervals from mice brains 20 days after injection. Exosomes were observed in most brain sections (approximately 12–13 brain sections, Supplementary Figure S1). In the hippocampus, exosomes were concentrated in the CA3 region of mouse hippocampal slices, very little were observed in CA1 or CA2 of the hippocampus (Figure 2D). Meanwhile, the control group showed little red fluorescence in the hippocampus and cortex at 3, 6 and 20 days after injection (Figure 2E, Supplementary Figure S2). Yuyama et al. (2014) continuously injected exosomes intraventricularly for 14 days and found significant reductions in Aβ levels and Aβ-associated synaptotoxicity in the mouse hippocampus, as well as reductions in Aβ deposition.

### Exosomes Target Microglia Preferentially and Stably in Mouse Brains

**Figure 3:**
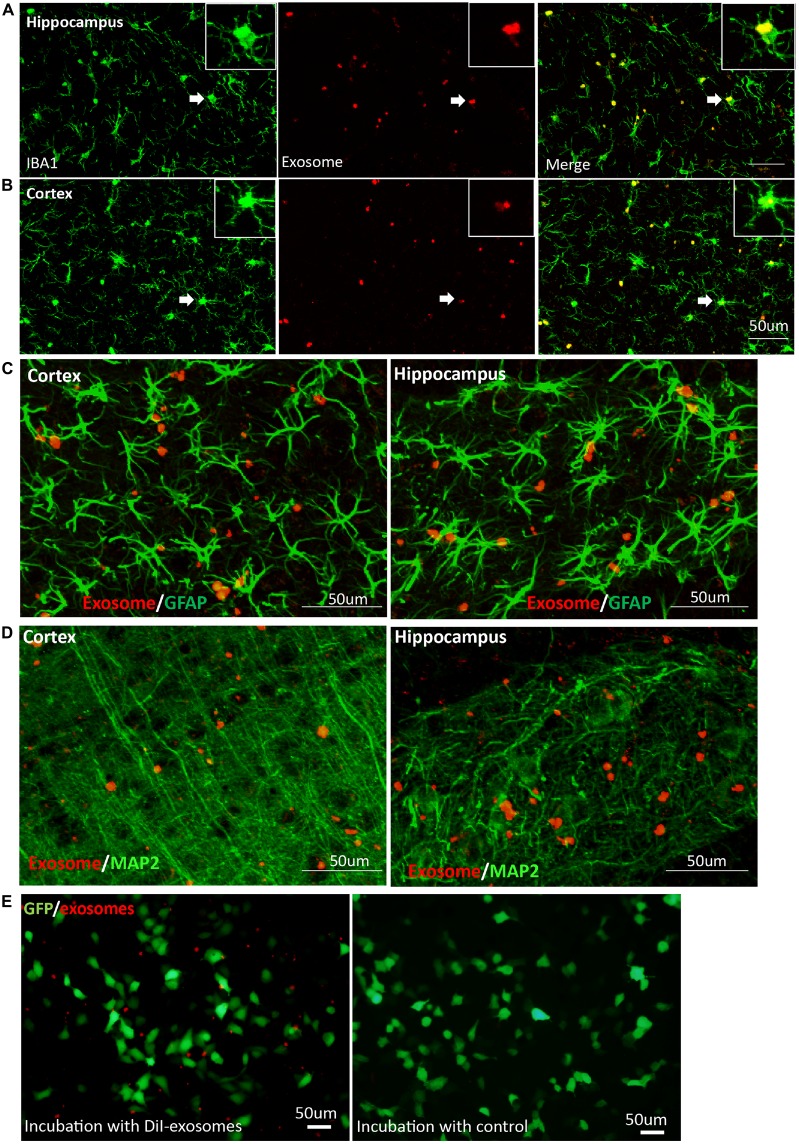
**Exosomes target microglia preferentially and stably.** Images were captured at 20 days after DiI-exosome injection in non-transgenic mice (17 months old). **(A,B)** Microglia in the hippocampus and cortex were stained with anti-Iba1 antibody. Merged figures show that the majority of exosomes are localized in microglia. Inset, higher magnification of an exosome-containing microglia cell labeled with Iba-1 antibody. **(C,D)** Exosomes (red) are not taken up by astrocytes labeled with anti- glial fibrillary acidic protein (GFAP) antibody (green, **C**) or neurons labeled with anti-MAP2 (green, **D**) in cortex and hippocampus. **(E)** SH-SY5Y cells were incubated with DiI-exosomes *in vitro*. After 24 h incubation, images were acquired using an inverted microscope. Compared with the control group (DiI incubation with PBS and being re-isolated, washed as the DiI-exosomes’ preparation and the same volume of control was incubated with sy5y cells), fluorescent signals are observed in the medium incubated with DiI-exosomes but are rarely detected in SH-SY5Y cells and cannot be detected in the control group.

As the resident immune cells of brain, microglia have been involved in brain injury and various neurological disorders. Recent studies show that oligodendrocyte-derived and N2a-derived exosomes were preferentially internalized by primary microglia *in vitro* (Fitzner et al., 2011). When exosomes were administered into mouse brain, microglia engulfment of exosomes was observed a few hours later. To further follow the fate of exosomes over a period of time in brain, we detected their presence in target cells on the 20th day after intracerebral injection. We observed that exosomes were still predominantly localized in Iba1-positive microglia in cerebral cortex and hippocampus (Figures 3A,B). In contrast, very little uptake of exosomes was observed in GFAP-positive astrocytes (Figure 3C) or MAP-2-positive neurons (Figure 3D) in cerebral cortex and hippocampus. Weak fluorescent signals could be occasionally detected in neurons of the DG, suggesting limited neuronal uptake of exosomes. Accumulation of exosomes may occur in the hippocampal CA3 region along the projection from the DG to the CA3 region (Figure 2D). We next used the neuroblastoma cell line SH-SY5Y with DiI-exosomes *in vitro*. After 24 h incubation with labeled exosomes, fluorescent signals were observed in the medium but rarely detected inside SH-SY5Y cells (Figure 3E). Combined with previous experimental results (Fitzner et al., 2011; Yuyama et al., 2012), our data demonstrated that exosomes target microglia preferentially and stably in mouse brains.

### Aging Does Not Affect the Phagocytosis of Exosomes by Microglia

**Figure 4:**
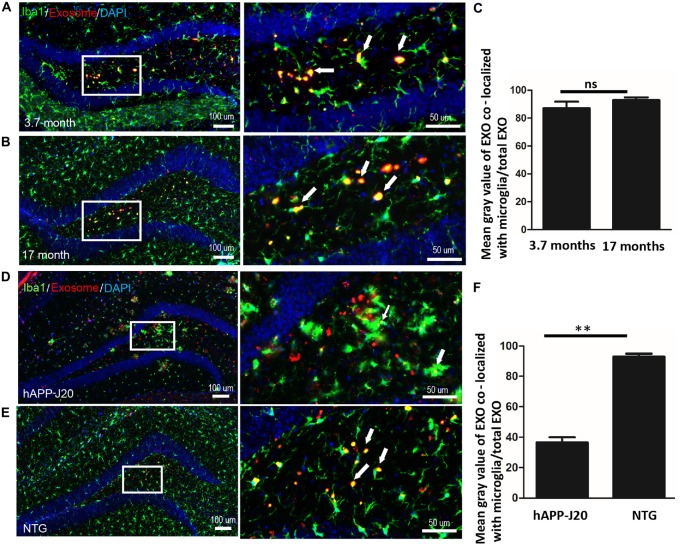
**Aging does not affect the phagocytosis of exosomes by microglia DiI-exosomes were stereotactically injected into the hippocampus.** Hippocampal immunofluorescence staining was detected after 20 days. **(A,B)** Hippocampal sections from 3.7 **(A)** and 17 **(B)** month old mice were stained with anti-Iba1 antibody (green). **(C)** Quantification of the relative uptake of exosomes by microglia (mean gray value of exosomes co-localized with microglia/total exosomes) in hippocampus of 3.7 and 17 months old mice (double-blinded, ImageJ analysis). ns, no statistically difference. EXO, exosomes. **(D,E)** Exosomes target microglia in hAPP-J20 mice with lower efficiency. Hippocampal sections from hAPP-J20 transgenic mice **(D)** and non-transgenic mice **(E)** were stained with anti-Iba1 antibody. **(F)** Quantification of the relative uptake of exosomes by microglia in hippocampus of hAPP-J20 transgenic mice and non-transgenic mice. *N* = 3 (5 brain slices for each mouse); **P < 0.01, two-tailed student’s *t* test was used.

Age-associated microglial senescence in the brain leads to abnormal function and may eventually promote neurodegeneration (Luo et al., 2010). Since cellular internalization of exosomes occurs through phagocytosis in microglia, we investigated whether aging could affect the ability of microglia to engulf exosomes. We compared phagocytosis of exosomes by microglia in the hippocampus between young (3.7 months old) and old (17 months old) mice. No significant difference was observed (Figures 4A–C, Supplementary Figure S3).

### The Ability of Microglial Engulfment of Exosome in AD Transgenic Mouse Brain Is Reduced

Several studies revealed that microglial phagocytic capacity is impaired in AD (Hickman et al., 2008). In this study, we compared microglial phagocytosis of exosomes in hippocampus between 17 month-old hAPP-J20 mice and littermate controls. We observed that microglial phagocytosis of exosomes was lower in hAPP-J20 mice (Figures 4D–F, Supplementary Figure S4).

### Exosomes Cluster Around the Aβ Plaques and Are Engulfed by Activated Microglia Nearby

**Figure 5:**
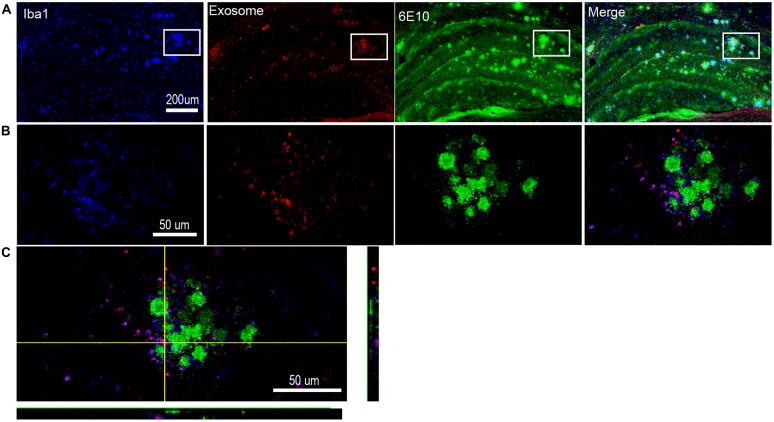
**Exosomes cluster around the Aβ plaques and are engulfed by nearby activated microglia. (A)** Representative photomicrographs showing the distribution of DiI-exosomes in hippocampus of hAPP-J20 transgenic mice. Microglia are labeled with anti-Iba1 (blue), exosomes are labeled with DiI (red) and anti-6E10 is used to label Aβ plaques (green). **(B)** Higher magnification images of the boxed areas in **(A)** show a single Aβ plaque. Exogenously injected exosomes and microglia cluster around the Aβ plaque. Moreover, the exosomes are largely localized in this microglia. **(C)** Representative confocal image through a single plane of exosome (red) internalized by microglia (green) in the hippocampus.

One pathological feature of AD is extracellular amyloid deposition and the presence of senile plaques (Beyreuther and Masters, 1991). Continuous intracerebral injection of neuroblastoma-derived or neuronal exosomes into AD transgenic mice results in marked reduction in Aβ pathology (Yuyama et al., 2014, 2015). However, injection of the astrocyte-derived exosomes into the brains of 10-day-old 5XFAD mice stimulates aggregation of Aβ *in vivo* (Dinkins et al., 2014). Here, we injected plasma exosomes into 17-month old AD mice to assess the relationship between plasma exosomes and Aβ plaques. We observed that exosomes clustered around the Aβ plaques, especially the large plaques (Figure 5). It has been demonstrated that extracellular Aβ plaques are often surrounded by activated microglia in both humans with AD (Styren et al., 1990) and AD animal models (Frautschy et al., 1998). We confirmed this phenomenon in hAPP-J20 mice (Figure 5). It is interesting that most exosomes clustered around Aβ plaques were localized in activated microglia (Figure 5), suggesting that microglia may play a role in AD pathogenesis through engulfing exosomes.

## Discussion

Neurodegenerative diseases, including AD, begin with dysfunction in a discrete region, and involve much larger areas of the brain at later stages. AD pathology has been proposed to spread through functionally and anatomically connected brain regions (Braak and Braak, 1991; Buckner et al., 2005; Braak et al., 2006; Harris et al., 2010), perhaps by a prion-like mechanism (Eisele et al., 2009; Frost and Diamond, 2010; Acquatella-Tran Van Ba et al., 2013; Marciniuk et al., 2013). Previous studies have demonstrated that exosomes facilitate the unique transmissible nature of prions (Vella et al., 2007; Guo et al., 2016). Many proteins associated with AD including APP and the APP-processing products, C-terminal fragments and Aβ can be found in exosomes from both neuronal cell cultures and brain tissues (Rajendran et al., 2006; Vingtdeux et al., 2007; Perez-Gonzalez et al., 2012). Pathogenic proteins involved in other neurodegenerative disorders also have been shown to be released by cells in association with exosomes (Bellingham et al., 2012).

In our study, we observed that exosomes diffused from the injection site in the DG to other brain regions, which could indicate the potential for exosome-bound pathogenic proteins to travel great distances in the brain. Previous studies have also shown that exosomes can cross the blood-brain barrier. When combined with our result that plasma exosomes can spread in the brain parenchyma, these data suggest that exosomes from peripheral blood may communicate with the CNS in physiological and pathological conditions. In addition, we found that exosomes concentrated in the CA3 region of the hippocampus. In contrast, very few exosomes were observed in the CA1 and CA2 areas of the hippocampus. This may due to projections from the DG to CA3 region. Synaptic connections between the mossy fibers of the granule cells in the DG and CA3 neurons, and the Schaffer collaterals from CA3 to CA1 neurons constitute the forward hippocampal polysynaptic circuit in mice (Harris et al., 2010). Further studies are needed to explore the relationship between exosomal diffusion and axonal projections in the hippocampus. It is reported that approximately 98% of all potent drugs that may be therapeutic for many neurological diseases in the CNS failed in clinic trials because of their inability to cross the blood-brain barrier (Pardridge, 2012). Exosomes have been increasingly used as delivery platforms, encapsulating reagents or siRNAs (Alvarez-Erviti et al., 2011; Haney et al., 2015). Therefore, the exosomal diffusion in brain observed in our study further supports the feasibility of using exosomes as a delivery system from the peripheral circulation to the brain.

Our data are consistent with previous observations that oligodendrocyte-derived exosomes are specifically and efficiently taken up by microglia (Fitzner et al., 2011). Harris et al. (2010) reported that oligodendrocyte-derived exosomes are selectively taken up by microglia via micropinocytosis; Yuyama et al. (2012) further showed that microglia engulf exosomes in a phosphatidylserine-dependent manner. In agreement with these previous studies, we found that plasma exosomes were efficiently taken up by microglia. Unlike short-term tracking in a previous report, we investigated the diffusion of exosomes in the brain for up to 20 days. Our results showed that exosomes can spread stably in the brain and are largely taken up by microglia. A portion of exosomes not engulfed by microglia were observed in the cortex and hippocampus, which rules out the possibility that the spreading of exosomes is due to the migration of microglia containing labeled exosomes. To evaluate whether neurons and astrocytes also engulf exosomes, we tested fluorescently-labeled exosomes in these cells. In contrast to microglia, very little uptake of exosomes was observed in astrocytes or neurons in cerebral cortex or hippocampus, which is supported by *in vitro* results (Fitzner et al., 2011).

The proteins and nucleic acids carried by exosomes play important roles in signal delivery and material exchange between cells. In a similar fashion, microglia may communicate with the other cells in brain through exosomes. However, exosomes can potentially carry pathogenic proteins, such as prions, propagating their toxic assemblies and promoting the progress of diseases (Vella et al., 2007; Guo et al., 2016). Engulfment of exosomes by microglia may prevent the propagation of exosome-bound pathogenic proteins to other cells. Interestingly, insulin-degrading enzyme and Aβ-degrading enzymes have been found in the exosomes secreted by microglia (Tamboli et al., 2010). Therefore, exosomes may enhance the ability of microglia to clear pathogenic proteins. Further studies are required to clarify the roles of exosomes in the CNS and their specific relationships with microglia.

Aging effects on microglial phagocytosis has been widely studied. A recent report suggests that the ability of primary murine microglia to take up exosome-associated oligomeric α-synuclein is compromised in aged mice (Bliederhaeuser et al., 2016). Moreover, phagocytic deficits are found in human monocytes from elderly individuals (Bliederhaeuser et al., 2016). Our studies in mouse brain show that aging did not alter the ability of microglia to take up plasma exosomes, suggesting that dysregulation of microglia caused by aging may not influence the engulfment of plasma exosomes.

Impaired microglial phagocytic capacity has also been observed in neurodegenerative diseases such as AD. Here we show that exosomes targeted microglia in hAPP-J20 mice with lower efficiency compared with control littermates. Microglia pro-inflammatory activation and dysfunction has been observed in AD brains. However, both the decreased amyloid-clearing ability of microglia (Flanary, 2005) and the damage to microglia by amyloid (Korotzer et al., 1993) have been reported in AD brain. von Bernhardi (2007) proposed that AD is caused by dysfunctional microglia rather than by hyperactive microglia. Microglia from aged PS1-APP mice have a 2–5 fold decrease in the expression of the Aβ-binding scavenger receptors RAGE, scavenger receptor A and CD36 compared to their littermate controls (Hickman et al., 2008), which indicates microglial dysfunction in AD and supports the idea that microglia senescence contributes to the pathogenesis of AD. Therefore, the observation in our study that engulfment of exosomes is reduced in the AD mouse model may due to phagocytic deficits of microglia in AD.

Recently, *in vitro* studies demonstrate that a fraction of Aβ peptide is released in association with exosomes (Rajendran et al., 2006). Exosomes enhance conformational changes in Aβ to form nontoxic amyloid fibrils and promote the uptake and clearance of Aβ by microglia (Yuyama et al., 2012). These findings suggest that exosomes are involved in Aβ metabolism in the brain. Here, we observed that exogenously injected exosomes clustered around Aβ plaques, especially large plaques, in hAPP-J20 mice. Interestingly, exosomal markers are found to be enriched in amyloid plaques in the brains of Tg2576 mice (Kokubo et al., 2005) and postmortem human AD patients (Rajendran et al., 2006). Meanwhile, activated microglia were found to surround the extracellular Aβ plaque as previously reported (Rogers et al., 1988; Frautschy et al., 1998; Stalder et al., 1999; Buckner et al., 2005). The confocal images show that most of the exosomes around the plaques are localized in these activated microglia. Yuyama et al. (2014, 2015) reported that glycosphingolipids are abundant in exosomes and Aβ can bind to the exosome surface via the glycan moieties of glycosphingolipids. Exosome-bound Aβ is then transported into microglia for degradation, resulting in a decrease in Aβ levels, Aβ plaques and Aβ-related pathologies in APP mice. Combined with the observations in our study, these data suggest that exosomes may play a role in trafficking of Aβ aggregates through microglia during disease progression. However, injection of astrocyte-derived exosomes into the brains of 10-day old 5XFAD mice stimulated aggregation of Aβ in at the site of injection (Dinkins et al., 2014). Increasing serum exosome content by treating 5XFAD mice with ceramide ultimately enhanced plaque formation (Dinkins et al., 2015) The size of amyloid plaques changes over days in brains of AD model mice (Bolmont et al., 2008). Whether the plasma exosomes support this change needs further exploration. It is worth noting that although engulfment of exosomes by microglia is inefficient in AD transgenic mice overall, the activated microglia clustering around plaques can take up exosomes effectively. This may indicate that phagocytosis in microglia clustering around plaques is stronger than other parts of the brain. Whether the plasma exosomes can decrease Aβ plaques and play a role in pathological process of AD, as well as which type of cells these functional exosomes come from, needs further investigation.

Overall, our study described the movement of exosomes across great distances in the brain, which can help to advance medical research of exosomes in neurodegenerative diseases. Moreover, exosomes may play roles in amyloid deposition through microglia in AD.

## Author Contributions

BZ, BS and TZ conceived and designed the study; TZ, JP, YC, YM, ZG, HP, LZ and HZ performed the experiments; JP and TZ processed and analyzed all data; BS and BZ wrote the manuscript with input from TZ. All authors have read and approved the final version of the manuscript.

## Conflict of Interest Statement

The authors declare that the research was conducted in the absence of any commercial or financial relationships that could be construed as a potential conflict of interest.
