# Supplementary material for: Plasma Exosomes Spread and Cluster Around β-Amyloid Plaques in an Animal Model of Alzheimer’s Disease
Source: Front Aging Neurosci. 2017 Feb 1;9:12. doi: 10.3389/fnagi.2017.00012 (PMC5285341; doi:10.3389/fnagi.2017.00012)
Supplement: Supplementary file 1 [file Image_1.pdf]

Plasma Exosomes Spread and Cluster Around β-Amyloid Plaques in an Animal Model of Alzheimer's Disease

Tingting Zheng[1], Jiali Pu[1], Yanxing Chen[1], Yanfang Mao[1], Zhangyu Guo[1], Hongyu Pan[2], Ling Zhang[2] ,Heng Zhang[2],Binggui Sun[2*], Baorong Zhang[1*]

[1]Department of Neurology, Second Affiliated Hospital, School of Medicine, Zhejiang University, 88 Jiefang Road, Hangzhou 310009, China

[2] Department of Neurobiology, School of Basic Medical Sciences, Key Laboratory of Medical Neurobiology (Ministry of Health of China), Key Laboratory of Neurobiology of Zhejiang Province. Zhejiang University School of Medicine, Hangzhou, Zhejiang Province 310058, China

*Correspondence: brzhang@zju.edu.cn(Baorong Zhang).

bsun@zju.edu.cn(Binggui Sun).

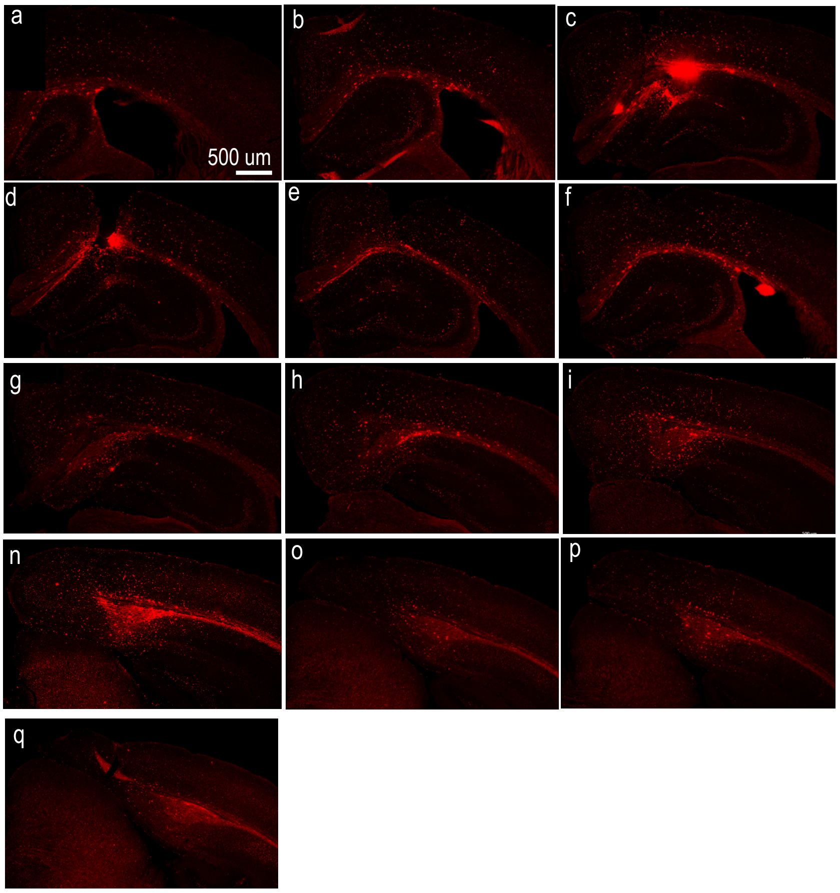

Figure1. Exosomes are widely distribute in brain Images were captured at 20 days after injection of exosomes in non-transgenic mice (a-q). 13 coronal brain sections that is about 200um between each section. Exosomes (red) are widely distribute in most brain sections.

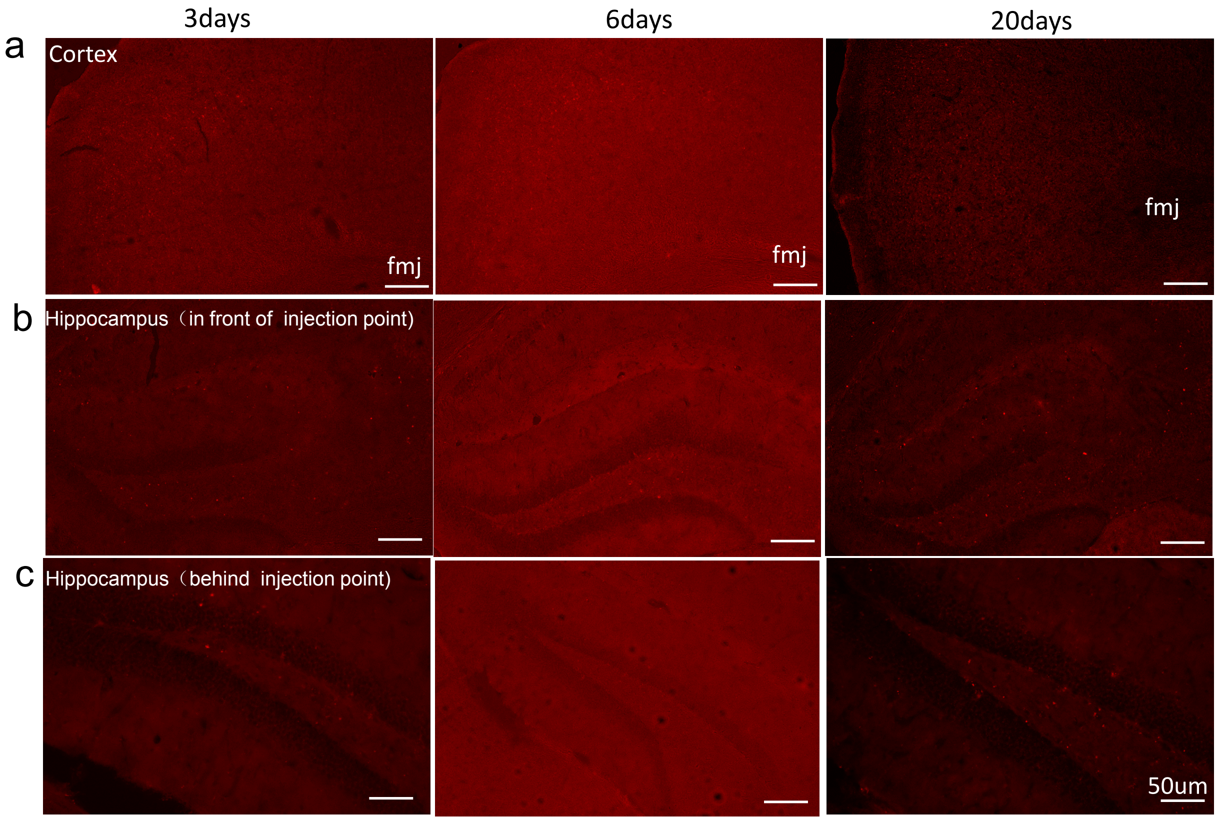

Figure2. The control (DiI incubation with PBS and being re-isolated, washed, re suspended in sterile PBS for three times as the DiI-exosomes' preparation) were injected into hippocampal DG region. Representative photomicrographs showing images captured at 3,6,20 days after injection

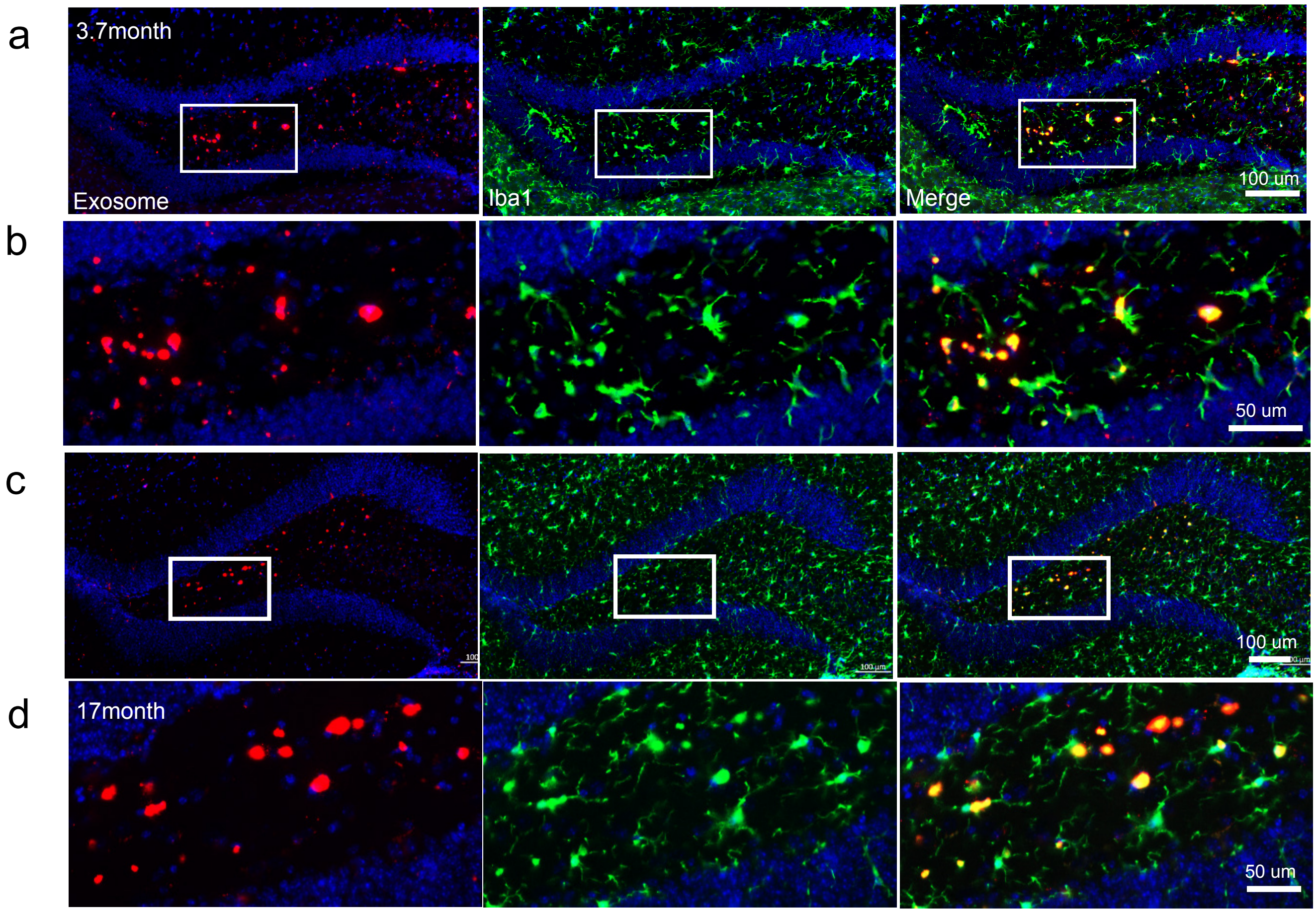

Figure3. **Aging did not affect the phagocytosis of exosomes by microglia** Red fluorescence-labeled exosomes were stereotaxically injected into the hippocampus. (a,c Hippocampal sections from 3.7 and 17 months old mouse were stained with anti-Iba1 antibody (green). The merge images show the exosomes taken up by microglia (b,d) higher magnification images of the boxed area in images a,c.

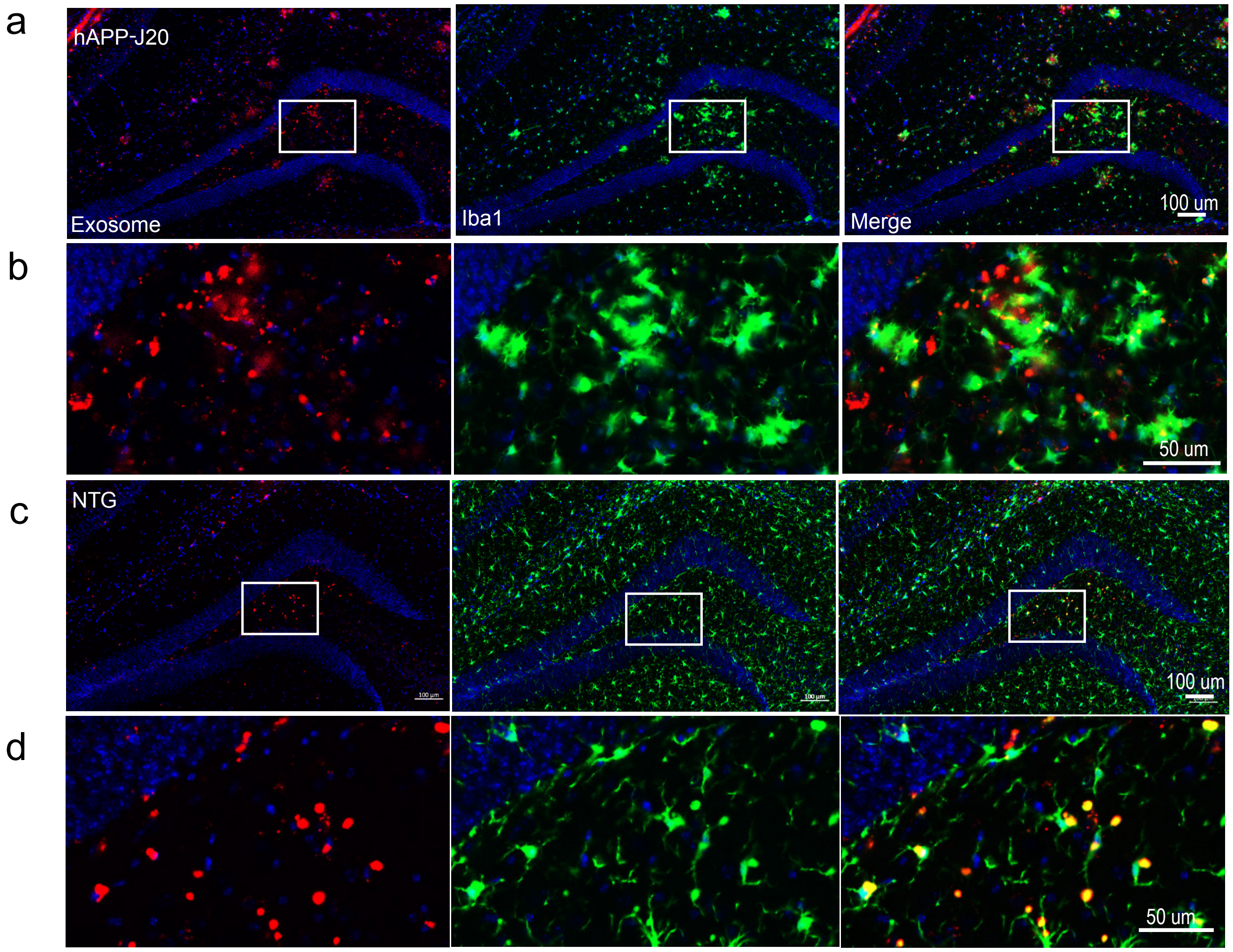

Figure4. Exosomes target microglia in hAPP-J20 mice with lower efficiency Red fluorescence-labeled exosomes were stereotaxically injected into the hippocampus. (a,c Microglia in hippocampal sections from hAPP-J20 mouse and NTG mouse are labeled with anti-Iba1 antibody. The merge images show the exosomes taken up by microglia. (b,d) higher magnification images of the boxed area in images a,c.
